# Cough-Anal Reflex May Be the Expression of a Pre-Programmed Postural Action

**DOI:** 10.3389/fnhum.2017.00475

**Published:** 2017-09-27

**Authors:** Paolo Cavallari, Francesco Bolzoni, Roberto Esposti, Carlo Bruttini

**Affiliations:** Human Motor Control and Posture Lab, Section Human Physiology of the Department of Pathophysiology and Transplantation, Università degli Studi di Milano Milan, Italy

**Keywords:** cough-anal reflex, intra-limb APAs, inter-limb APAs, common mechanism, cough, external anal sphincter

## Abstract

When coughing, an involuntary contraction of the external anal sphincter occurs, in order to prevent unwanted leakages or sagging of the pelvis muscular wall. Literature originally described such *cough-anal* response as a *reflex* elicited by cough, therefore identifying a precise cause-effect relationship. However, recent studies report that the anal contraction actually precedes the rise in abdominal pressure during cough expiratory effort, so that the sphincter activity should be *pre-programmed*. In recent years, an important family of pre-programmed muscle activities has been well documented to precede voluntary movements: these anticipatory actions play a fundamental role in whole body and segmental postural control, hence they are referred to as *anticipatory postural adjustments* (APAs). On these basis, we searched in literature for similarities between APAs and the cough-anal response, observing that both follow the same *predictive homeostatic principle*, namely that anticipatory collateral actions are needed to prevent the unwanted mechanical consequences induced by the primary movement. We thus propose that the cough-anal response also belongs to the family of pre-programmed actions, as it may be interpreted as an APA acting on the abdominal-thoracic compartment; in other words, the cough-anal response may actually be an *Anticipatory Sphincter Adjustment*, the *visceral counterpart of APAs*.

## Introduction

The *cough-anal reflex* has been described as the contraction of the external anal sphincter occurring when coughing (Meagher et al., 1993). Literature is however unclear in discriminating whether the action on the sphincter is a reflex response due to the abdominal and pelvic floor dynamics, or an integral component of the cough itself. According to the past studies, any maneuver causing an increase in abdominal pressure, like cough, Valsalva etc., determine a reflex contraction of perineal muscles (Parks et al., 1962; Bors, 1966; Deindl et al., 1993; Bø and Stien, 1994). Some authors even provided evidences that during voluntary cough the external anal sphincter is recruited after intercostals and abdominal muscles, suggesting that this response is mediated by a polysynaptic reflex (Chan et al., 2004). In both these hypotheses, a precise cause-effect relationship is identified, in which the reflex contraction of the sphincters prevent unwanted leakages or sagging of the muscular wall of the pelvis. On the contrary, recent literature reports that the sphincters contraction actually precede the rise in abdominal pressure during cough expiratory effort (Constantinou and Govan, 1982; Thind et al., 1990; Thind and Lose, 1992), also showing that the increase in anal or vaginal closing pressure precedes the cough motor act (Sapsford and Hodges, 2001) and that the external anal sphincter is recruited in advance of intercostals muscles (Deffieux et al., 2006). Based on their findings, Sapsford and Hodges (2001) and Deffieux et al. (2006) forwarded the alternative idea of a *pre-programmed* central control of the external anal sphincter activity for granting continence during cough.

From another perspective, if one examines the abdominal-thoracic compartment, a forceful increase of the internal pressure may be induced both by the respiratory muscles (diaphragm relaxation and internal intercostal muscles contraction) which promote the compression of the thoracic cavity, and/or by the abdominal wall muscles (contraction of rectus abdominis, transverse abdominis, external oblique muscle and internal oblique muscle) which press the abdominal organs cranially, reducing the volume of the thoracic cavity to help expel air, vomit, urine and feces from the body (Iscoe, 1998).

A different temporal and spatial recruitment of the wall muscles and of sphincters may thus lead to different behavioral results: expulsion of the air during speaking, singing, yelling or coughing (in this case perineal muscles and perineal sphincters should contract in synergy); vomit, when abdominal pressure increases and the lower esophageal sphincter is relaxed; defecation and micturition, when perineal muscles are contracted, to stiffen the pelvic floor, while perineal sphincters are relaxed, to offer an escape route to excretions. Not to mention the complex mechanism of foetal delivery, to which cooperates the uterine contraction.

Returning to the *cough-anal reflex/response* question, an important family of *pre-programmed* muscle contractions has been well documented when studying voluntary movements: these anticipatory adjustments play a fundamental role in whole body and segmental postural control (Bouisset and Do, 2008; Cavallari et al., 2016), so that they are referred to as anticipatory postural adjustments (APAs). By analogy, we propose that also the sphincter control may belong to the family of the *pre-programmed actions*, and in this view, the cough-anal reflex may be interpreted as an APA on the thoraco-abdominal compartment.

## Anticipatory Postural Adjustments and Voluntary Movements

In humans, there are several examples of *pre-programmed*, *anticipatory* actions which accompany a willed movement. Indeed, APAs are usually described as unconscious muscular activities preceding the primary movement and aim to counterbalance the perturbation caused by it (for a review, see Massion, 1992; Bouisset and Do, 2008). APAs are so intimately linked to the motor program that the involuntary neural command to postural muscles is shared with the voluntary command to prime mover muscles (Bruttini et al., 2014). The importance of APAs is apparent when considering motion of one single limb (e.g., Belen’kii et al., 1967 for the upper limb; Alexeief and Naidel, 1972 for the lower limb), in this context, the main goal of APAs is to minimize the changes in the body center of mass, to keep its projection within the support area, and to counteract the self-initiated postural perturbation. APAs also contribute to initiate the displacement of the body center of mass when starting gait (Brenière et al., 1987) or whole body reaching movements (Stapley et al., 1998, 1999). These activities, called *inter-limb APAs*, set one or more fixation chains spreading over several muscles of different limbs. APAs may also precede movements of tiny masses, like a brisk flexion of the index-finger when performed in a seated position (Caronni and Cavallari, 2009). In this case, they are named *intra-limb* APAs as the fixation chain is limited to muscles acting on proximal joints (Cavallari et al., 2016). Taking into account the small mass of the moving segments, and the fact that this movement cannot affect the whole-body balance, the role of *intra-limb* APA has been attributed to the precision of the final movement (Bruttini et al., 2016). Note also that despite the different aim, *inter-* and *intra-*limb APAs share many behavioral properties: they are distributed to muscles of the same limb in which the movement occurs, precede the onset of the voluntary movement, are polarized according to the task direction in space, are scaled to the amplitude of the perturbation and adapt to changes in the postural requirement of the task (Cavallari et al., 2016).

## Cough-Anal Reflex

### Cough

Cough is a defensive reflex, triggered by sensory inputs arising from the airways, that generates a high velocity air flow so as to free the respiratory tract from whichever obstruction (for a review, see Ando et al., 2014). It consists of a modified respiratory act which starts with a profound preparatory inspiration phase, followed by a brief compressive phase, in which an expiratory effort is exerted against the closed glottis, and finally by an expulsive phase in which a sudden opening of the glottis is coupled to a quick and strong expiratory muscles contraction (Fontana and Lavorini, 2006).

Cough reflex, is aimed at protecting the respiratory tract from irritation agents, foreign bodies and microorganisms, either inhaled or *in loco* produced (Bolser and Davenport, 2002; Bessac and Jordt, 2010), such action escapes any voluntary control and its processing seems to be entirely based on brainstem structures (Baekey et al., 2001; Canning and Mori, 2010). Nevertheless, more and more literature reports that not only sensory and motor but also cognitive and affective mechanisms may play a role in controlling cough, being able either to promote or inhibit it (Widdicombe, 1995; Fong et al., 2004; Mazzone et al., 2007, 2011; Davenport, 2009). Cough, like most respiratory maneuvers, can be also initiated at will, indeed cough or cough-like maneuvers are quite common in non-verbal communication, e.g., to require attention or express disagreement, to pretend to be ill or underline the symptoms of a real disease.

Reflex and voluntary cough present important differences in several aspects. Apart from the fact that the former is triggered by a precipitating sensory stimulus while the latter is not, both types of cough require similar muscle groups, but voluntary coughing recruit such muscles according to a precise sequential order (Lasserson et al., 2006; for review see Magni et al., 2011). Moreover, the peak intra-abdominal pressure reached during reflex cough is higher than that reached during voluntary cough (Addington et al., 2008).

Several studies aimed at identifying the CNS structures that govern coughing and their neural interconnections. Although an extensive list of candidate structures have been proposed, detailed studies about the role of each structure are still missing, so that specific aspects such as the relative inter-structure connections and the temporal organization are not yet fully determined. Some recent insight has been provided by McGovern et al. (2012a,b), who used neurotropic viruses to map the interconnections between neuronal networks.

The sensory fibers of the vagal nerve than stem from the airways project to the trigeminal sensory and solitary tract nuclei. In turn, these nuclei project to the hypothalamus and to the parabrachial nuclei, to the thalamus and to subthalamic nuclei, as well as to the amygdala and up to several cortical areas, which include the somatosensory cortices as well as the cingulate, insular and orbital cortices (McGovern et al., 2012a). These data allowed Mazzone et al. (2013) to propose that two ascending pathways originate from the airways: the first that projects to the ventral and basal portion of the thalamus and then up to the somatosensory cortices, while the second crosses the medial and dorsal thalamus, reaching the insula, as well as the orbital and cingulated cortices.

Important results about the brainstem circuitry governing reflex cough have been obtained by employing pharmacological and electrophysiological techniques on deeply anesthetized or even decerebrate animals. Indeed, such experiments showed that the afferent inputs to the pontine and medullary nuclei are able to trigger a reconfiguration of the respiratory central pattern generator, changing its activity from the classical motor pattern that produce breathing to the specific pattern driving cough (Baekey et al., 2001, 2003; Canning et al., 2004; McGovern et al., 2012a). However, an in depth description of the brainstem mechanisms that control cough in the human being is still lacking.

In the human being, urge-driven cough may be not exclusively governed by brainstem reflex circuitries, as other hierarchically higher brain areas, such as the insular, cingulate and somatosensory cortices, may provide an important contribution (Mazzone et al., 2011). Even if the exact role of these cortical areas is still unclear, it may be conceived that they take part in controlling the accessory muscles in cough, as well as in arranging the voluntary control of cough. In support of such a proposal, Shima et al. (1991) reported that in monkeys the stimulation of the posterior insular cortex is able to induce contractions in the contralateral muscles. Similarly, the mid-cingulate cortex has been reported to be active when voluntarily coughing, and also during a voluntary sniff or breath (Simonyan et al., 2007; Mazzone et al., 2011). Finally, it is noteworthy that cough-like actions may be elicited in the anesthetized cat by stimulating the amygdala or the suprasylvian gyrus with electrical pulses, and that the reflex-cough, triggered by stimulating the superior laryngeal nerves (afferent components), is inhibited if a simultaneous stimulus is applied to the orbital or cingulate gyri (Kito et al., 1977; Kasé et al., 1984).

### Pelvic Floor Muscles

Pelvic floor muscles are involved in the function of the lower urinary tract and lower digestive tract, as well as in sexual functions. Thus, their neural control, which is somatic in origin, must consider their involvement in visceral activity. A coordination with the autonomic motor nervous systems should then be previewed.

The function of pelvic floor/sphincter lower motor neurons is organized differently from other groups of motor neurons. In contrast with the reciprocal innervations, commonly observed in limb muscles, the neurons innervating each side of the pelvic floor must work in harmony and synchronously, as a functional unit. As an example, in continent women bilateral pubococcygei muscles contract simultaneously and so do both halves of the sphincter muscle (Deindl et al., 1993). But flexible activation patterns could be possible, due to the unilateral innervation of these muscles. Indeed, Kenton and Brubaker (2002) reported that levator ani and urethral sphincter may show differences in activation patterns. On voiding, the external urethral sphincter should relax, preceding the detrusor contraction, while the anal sphincter is tonically active; on the contrary, both sphincters relax when defecating (Read and Sun, 1990). It is also known that a voluntary inhibition of the urethral sphincter may be achieved even without actually voiding (Sundin and Petersén, 1975).

Reflex activity may be triggered by an increase of intra-abdominal pressure or by a distension of pelvic organs, but it cannot be excluded that long-loop pathways activated by noxious stimuli may contribute as well (McMahon et al., 1982). It has been also reported that reflex activity may be modulated by inhibitory descending pathways from the brainstem and the motor cortex (Mackel, 1979). In particular, the descending inhibitory projections from pontine tegmentum, via the commissural nucleus, seem to be crucial for a correct timing of the sphincter relaxation during voiding (Blok and Holstege, 1998). Importantly, the complex motor sequence which drives bladder and sphincter activity is also accompanied by coordinated postural changes (Stafford et al., 2012).

In addition, pelvic floor muscles have been shown to contribute to both postural and respiratory functions (Hodges et al., 2007). These authors indeed demonstrated that during voluntary arm movements the activation of Anterior Deltoid is preceded by EMG activity in pelvic floor muscles; these muscles are activated before the intra-abdominal pressure increases, also contributing to stiffen the sacroiliac joints, therefore such activity should actually be an APA. Hodges et al. (2007) also recorded pelvic floor muscles activity during quiet breathing and under increased dead-space condition, reporting that such activity was tailored according to the respiration phase, increasing together with abdominal muscles activity; a finding that suggests the presence of *visceral* APAs when breathing. Finally, it is important to note that when discussing the above results, these authors concluded for a pre-programmed control, in view of the observation (Constantinou and Govan, 1982) that during a cough the increase in urethral pressure precedes by ~200 ms the increase in bladder pressure.

### Cough and Pelvic Floor Muscles Activity

According to the past literature, cough, Valsalva maneuver, and more generally, abdominal distension, determine a reflex contraction of perineal muscles (Parks et al., 1962; Bors, 1966; Deindl et al., 1993; Bø and Stien, 1994). In fact, cough determines an increase in abdominal pressure and subsequently an increase in pressure in the bladder and the rectum. But every increase in bladder or rectum pressure requires a simultaneous increase of contraction of the sphincters to prevent leakages or the sagging of the muscular wall of the pelvis. Thus, a contraction of striated urethral muscle or striated anal sphincter is required. Many electrophysiological studies have described these actions (Parks et al., 1962; Deindl et al., 1993; Meagher et al., 1993; Bø and Stien, 1994), defining them as *reflex responses*. In this regard, Chan et al. (2004) showed that the external anal sphincter is recruited after intercostals and rectus abdominis muscles, both during voluntary cough and sniff. Moreover, the latency between rectus abdominis and external anal sphincter activation decreased as the cough effort increased. These authors concluded for a polysynaptic origin of the cough-anal reflex, but also reported that the afferent pathway for such late external anal sphincter response does not involve sensory input from the anal mucosa. Therefore they suggested that the reflex afferent pathway could come from muscle spindles or other sensory receptors in pelvic floor muscles, ligaments and fascia, as well as from the viscera or bladder. Unfortunately, Chan et al. (2004) did not recorded intra-abdominal pressure, which is the most probable cause of excitation for all such sensory receptors.

On the contrary, during voluntary cough, an increase in intra-urethral pressure precedes (by 100–240 ms) the rise in bladder pressure in healthy volunteers (Constantinou and Govan, 1982; Thind et al., 1990; Thind and Lose, 1992) and electromyographic (EMG) activity of the urethral sphincter occurs before cough in women suffering stress incontinence (Roskar et al., 1981; Heidler et al., 1987; Thüroff et al., 1987). These results fits with the hypothesis forwarded by van der Kooi et al. (1984) that the increase in abdominal pressure should follow the contraction of pelvic floor muscles.

More recently, Sapsford and Hodges (2001) reported that cough, which induce a rise in gastric pressure, is preceded by a rise in anal or vaginal pressure. These authors also underlined that this response should thus be *pre-programmed*. Moreover, Deffieux et al. (2006) reported that, during voluntary cough in continent women, external anal sphincter activity increased not only before intra-abdominal pressure but also before intercostal muscle activity (latency ranging from 40 ms to 800 ms), a result in apparent contrast with that of Chan et al. (2004). Deffieux et al. (2006) actually provided a detailed critic of those results, underlining that Chan’s group analyzed just one subject, did not record intra-abdominal pressure and used needle electrodes for EMG recording, which just record activity from small areas of muscles, while surface electrodes are better adapted to obtain accurate measurements of whole muscle activity. Therefore, also these authors concluded for a *pre-programmed* central control of the external anal sphincter activity aiming to maintain continence. In addition, it has been demonstrated that pelvic floor muscle contraction not only anticipates, but is also proportional to the rise in intra-abdominal pressure caused by the cough effort (Amarenco et al., 2005), a finding that replicates a well known behavior of the APAs.

Finally, although many authors suggest that anticipatory activity in pelvic floor muscles *cannot be a reflex response* to the afferent input generated by the stretch of the thoracic or abdominal muscles, since it precedes both voluntary and involuntary abdominal muscles recruitments, nobody explicitly states that the anticipatory pelvic floor response during cough may be *an anticipatory component of the cough itself* involving higher integrative centers. In this regard, it is interesting to note that the correlation between the *rectus abdominis* *to external anal sphincter latency* and the *cough effort* reported by Chan et al. (2004) is also consistent with the correlation between the *APAs* *to prime mover* *latency* and *movement speed* demonstrated by Horak et al. (1984).

## Ontogenesis of Postural and Sphincter Anticipatory Adjustments

The control of body position in space develops with different intensity during life span (Assaiante et al., 2005; Soberaa et al., 2011). As an example, Zaino and McCoy (2008) showed that young healthy children (6–8 years old) exhibit much higher variability of posture control than older healthy children (10–12 years old). It is also reported that the age 7–9 years is an important period of their life in which children master postural control (Massion, 1998). Moreover, Schmitz et al. (1999) showed that children 3–4 years old develop APA, although they show coexistence of both adult-like and immature patterns, concluding that this anticipatory activities are being set up and that children are progressively mastering them.

It is also well known that all children have wetting and/or soiling accidents at one time or another. Achievement of urinary continence is an important developmental step that most children attain with the assistance of their parents and caregivers, however literature reports that about 15%–20% of children become partially toilet trained but continue to have wetting accidents even after the age 5 (Issenman et al., 1999). The first awareness of bladder and rectal functions usually occur between one and two years of age. The neural mechanisms involved in the storage and periodic elimination of urine undergo marked changes during prenatal and postnatal development (de Groat, 2002; Jansson et al., 2005). In the first years of life, voiding is controlled by a primitive spinal reflex pathway. Voluntary control over striated muscle sphincter usually occurs by the age of 3 years (Sillén, 2004). As the human CNS matures, higher brain centers contribute in modulating reflex voiding (see Figure 7 of Fowler et al., 2008). In adults, injury or disease of the nervous system can lead to the re-emergence of primitive reflexes Geirsson et al., 1993; Jiang et al., 2002).

Changes in brain structure are continuous throughout life (Chugani et al., 1987; Giedd et al., 1996). By the age 2, the brain has reached 75% of its adult weight (Carmichael, 1990) and the processes of synaptic pruning and cell death are most active during these early years (Huttenlocher, 1979; Huttenlocher et al., 1982). During the school-age years, strong signs of brain maturation are appreciable, especially in its connectivity (Lebel et al., 2008). MRI measures of the structure in fibers tracts correlate with behavioral indices that also change in this period (Schmithorst and Yuan, 2010; Tamnes et al., 2010a,b). Later changes involve the associative neocortex, which continues to develop well into the third decade (Yakovlev and Lecours, 1967), and the corpus callosum, which connects all major subdivisions of the cerebrum (Pujol et al., 1993).

Thus, before school-age all kinds of anticipatory actions, preceding primary movements, seems to be immature, either because the whole system is not still developed or because a part of it is not properly functioning. The achievement of a complete action (anticipatory and willed; postural or non-postural), therefore requires repetition learning (Draganski et al., 2004) and memory (Takeuchi et al., 2010).

## Similarities between APAs and Cough-Anal Response

As shown above, APAs and cough-anal response share many common features: they are both anticipatory with respect to the primary movement, so that they cannot be *reflex* responses but must be *pre-programmed*; they both prevent unwanted mechanical consequences (postural perturbation or sphincter leaking); they are both scaled to the amplitude of the perturbation; and finally, they seem to follow the same ontogenetic steps.

Indeed, a conceptual similarity may be envisaged among inter-limb APAs, intra-limb APAs and the anal response. In fact, in all cases a single* predictive homeostatic principle* (Moore-Ede, 1986; Freddolino and Tavazoie, 2012) seems to be followed, namely that anticipatory collateral actions are needed to correctly perform the primary movement.

In the case of *inter-limb APAs*, that minimize the changes of the body center of mass and that counteract the self-initiated postural perturbation, this principle provides the maintenance of stability (equilibrium) of the whole body during an action (Massion, 1992; Bouisset and Do, 2008). These APAs spread over several muscles of different limbs, creating one or more long fixation chains.

In the case of *intra-limb APAs*, that maintain the stability of the different segments of a single limb, it means to successfully carry through a precise and coordinated movement (Caronni and Cavallari, 2009; Caronni et al., 2013; Bruttini et al., 2016; Cavallari et al., 2016). These APAs spread over several muscles of the same limbs, creating short fixation chains.

In the case of the *anal* or* urinary responses*, the anticipatory contraction of the sphincter permits to prevent fecal or urinary leakage during an increase of abdominal pressure, due to a sudden action. A behavior that should be defined *anticipatory adjustment* as well.

A final comment deserves the possible neural networks that may be involved in the control of postural and sphincter muscles. Indeed, some analogies may be directly outlined, while other may be actually tested. First, as mentioned in the “Cough” section, animal experiments showed that the afferent inputs to the pontine and medullary nuclei are able to trigger a reconfiguration of the respiratory central pattern generator, changing its *breathing* activity into a *cough* pattern (Baekey et al., 2001, 2003; Canning et al., 2004; McGovern et al., 2012a). In parallel, it has been shown that the *automatic gait* pattern of the locomotion central pattern generator may be influenced by pontine neurons (for a review, see Takakusaki, 2017). However, the advanced *pre-programmed* control of posture required for skilled and goal-directed movements involve many cortical areas, as well as basal ganglia and cerebellum (see Takakusaki, 2017). In this regard, the involvement of supplementary motor area and cerebellum in APAs control has been experimentally demonstrated by Bolzoni et al. (2015) and Bruttini et al. (2015). It would then be interesting to test if magnetic or DC stimulation of these structures would produce comparable effects on the anticipatory recruitment of postural muscles and of external sphincters, during both voluntary movements and respiratory/cough tasks.

## Conclusion

On these basis, it is proposed that a common pre-programmed (i.e., feed-forward) mechanism may govern several kinds of anticipatory actions. In fact, in all cases the brain seems to control in the same way the general *predictive homeostasis* of the body, accomplishing movements either voluntary- or urge-driven. Thus, *Anticipatory Sphincter Adjustments* are likely the *visceral counterpart of APAs*. Of course, this speculative conclusion is just an hypothesis, which needs further experimental testing. Indeed, it could be interesting to investigate the EMG activity of visceral muscles, such as pelvic floor muscles, during other movements like hiccupping or chest vs. diaphragmatic breathing, so as to ascertain if such activities are actually anticipatory also in these conditions. Other evidences may come from testing if stimulation of the supplementary motor area or the cerebellum would affect in parallel the activation of both postural muscles and external sphincters. Indeed such finding would strengthen the analogy between the cough-anal response and the APAs.

## Author Contributions

PC proposed the idea. PC and RE collected and critically analyzed the literature. All authors contributed in writing and discussing the manuscript. All authors approved the final version and agree to be accountable for all aspects of this work.

## Conflict of Interest Statement

The authors declare that the research was conducted in the absence of any commercial or financial relationships that could be construed as a potential conflict of interest.
